# PEG10 overexpression induced by E2F-1 promotes cell proliferation, migration, and invasion in pancreatic cancer

**DOI:** 10.1186/s13046-017-0500-x

**Published:** 2017-02-13

**Authors:** Yun-Peng Peng, Yi Zhu, Ling-Di Yin, Jing-Jing Zhang, Ji-Shu Wei, Xian Liu, Xin-Chun Liu, Wen-Tao Gao, Kui-Rong Jiang, Yi Miao

**Affiliations:** 1 Pancreas Center, First Affiliated Hospital of Nanjing Medical University, 300 Guangzhou Road, Nanjing, 210029 Jiangsu Province People’s Republic of China; 2 Pancreas Institute, Nanjing Medical University, Nanjing, 210029 Jiangsu Province People’s Republic of China

**Keywords:** Pancreatic cancer, PEG10, Progression, E2F-1

## Abstract

**Background:**

Overexpression of paternally expressed gene-10 (PEG10) is known to promote the progression of several carcinomas, however, its role in pancreatic cancer (PC) is unknown. We investigated the expression and function of PEG10 in PC.

**Methods:**

PEG10 expression and correlation with PC progression was assessed in cancerous tissues and paired non-cancerous tissues. Further, the role of PEG10 in PC cell progression and the underlying mechanisms were studied by using small interfering RNA (Si-RNA).

**Results:**

PEG10 expression was significantly higher in cancerous tissues and correlated with PC invasion of vessels and Ki-67 expression. Si-RNA mediated PEG10 knockdown resulted in inhibition of proliferation and G0/G1 cell cycle arrest, which was mediated by p21 and p27 upregulation. A decrease in PC cell invasion and migration, mediated by ERK/MMP7 pathway, was observed in PEG10 knockdown group. Further, findings of ChIP assay suggested that E2F-1 could directly enhance the expression of PEG10 through binding to PEG10 promoter.

**Conclusions:**

In conclusion, PEG10 was identified as a prognostic biomarker for PC and E2F-1 induced PEG10 could promote PC cell proliferation, invasion, and metastasis.

## Background

Pancreatic cancer (PC) is a highly invasive malignancy, which is the fifth leading cause of cancer-related deaths with a poor 5-year overall survival rate (<6%) [1]. Over the past few decades, there has been a marked increase in the incidence of PC [2]. Due to lack of obvious symptoms at the early stage of PC and a high rate of invasion of blood vessels, lymph nodes, and nerves, treatment through surgical resection is possible in less than 20% of PC cases [3]. Furthermore, pancreatic fibrosis and other factors lead to resistance to chemotherapy [4]. For these reasons, identification of novel therapeutic and prognostic biomarkers is a preferred approach for the development of diagnosis and treatment of PC. Several molecules have been recently reported to be crucial for therapy and prognosis of PC, such as MUC-4, LSD1, and FHL2 [5–7].

PEG10 (also known as EDR, HB-1, Mar2, MEF3L, Mart2, and RGAG3) is a paternally expressed imprinted gene which was first reported by Ono R et al. in 2001 [8]. PEG10 is expressed not only in brain, kidney, and lung tissues in adults but also in embryonic tissues, such as placenta [9]. Multiple functions have been attributed to this gene, for example, it participates in cell proliferation and differentiation [10, 11], and inhibition of cell apoptosis [12]. In addition, some studies have shown that PEG10 was generally overexpressed in several kinds of malignancies, such as lung cancer, hepatic cancer, and B-cell chronic lymphocytic leukemia [13–15]. The overexpression of PEG10 is significantly associated with the proliferation, progression, prognosis, and metastasis of such malignancies.

However, the levels and role of PEG10 in patients with PC have not been investigated extensively. Therefore, in the present study we have assessed the expression of PEG10 in pancreatic cancerous tissues and paired adjacent non-cancerous tissues by immunohistochemistry (IHC). We have also evaluated whether there is a correlation between PEG10 expression and clinicopathological features of PC patients as well as their survival rate. Further, the role of PEG10 in progression of PC cells was also evaluated.

## Methods

### Patients

PEG10 mRNA expression for 178 pancreatic cancer patients were downloaded from The Cancer Genome Atlas (TCGA) data portal (http://cancergenome.nih.gov/). A total of 206 formalin-fixed, paraffin-embedded tissues, including 103 cancerous tissues and 103 paired adjacent non-cancerous tissues were obtained from patients diagnosed with PC at Pancreas Center, The First Affiliated Hospital of Nanjing Medical University from 2005 to 2012. All patients received routine preoperative preparation and surgical therapy. The cancerous tissues and paired adjacent non-cancerous tissues were respectively integrated into relevant tissue microarray used for IHC using microarray punching instrument. Our study was approved by the Ethics Committee of the First Affiliated Hospital of Nanjing Medical University. Written informed consents were obtained from all patients undergoing surgery.

### Reagents and antibodies

Anti-human PEG10 antibody was obtained from Novus (Colorado, USA). Anti-human CDK4, CDK2, p21, p27, SKP2, and E2F-1 antibodies were purchased from Cell Signaling Technology (CST, Massachusetts, USA). Anti-human Cyclin E1 antibody was obtained from Abcam. Si-RNAs for PEG10 and E2F-1 and respective negative controls were purchased from GenePharma. Annexin V PE apoptosis detection kit was purchased from eBioscience (Hatfield, UK) and PI/RNase staining buffer was obtained from BD biosciences (New York, USA). Cell counting kit-8 (CCK-8) and EDU kit were obtained from Dojindo and RIBOBIO, respectively. Trizol reagent and PrimeScript RT Master Mix (Perfect Real Time) were both obtained from TaKaRa (Shiga, Japan) and FastStart Universal SYBR Green Master was purchased from Roche.

### Cell lines and cell culture

Pancreatic cancer cell lines AsPC-1, Mia PaCa-2, SW1990, BxPc-3, Capan-2, CFPAC-1, PANC-1 were available in our laboratory. Normal human pancreatic ductal cell line hTERT-HPNE was purchased from the American Type Culture Collection (ATCC; Rockville, MD, USA). Pancreatic cancer cell lines were cultured in DMEM supplemented with 10% FBS, penicillin (100 U/mL) and streptomycin (100 μg/mL). hTERT-HPNE cell line was cultured according to the recommendation of ATCC.

### IHC analysis

Immunohistochemical staining was performed using a standard immunoperoxidase staining procedure. The tissue sections were viewed independently by two experienced pathologists, who were blinded to the clinicopathological information and clinical outcomes of the patients enrolled in our study. The percentage of PEG10 positive cells was scored as follows: 0 (0%), 1 (1%), 2 (2%) …… 99 (99%), 100 (100%). The staining intensity was visually scored as follows: 0 (negatively stained), 1 (weakly stained), 2 (moderately stained), and 3 (strongly stained). Both the percentage of positive cancer cells and staining intensity were decided independently by double-blinded manner. IHC score for each case was calculated by the following formula: IHC score = positive rate score × intensity score. All patients were divided into three groups (high, medium, and low) according to the tri-sectional quantiles of PEG10 IHC score. And then, cases with IHC score >50 (high and medium) were regarded as high expression of PEG10; on the contrary, cases with IHC score ≤50 (low) were regarded as low expression.

### Small interfering RNA and plasmid vector related assays

Cells were seeded in 12-well plates (1.8 × 10^5^ cells/well) and cultured in complete medium for 12 h. After replacing fresh complete medium, Si-RNAs (or plasmid vector) and negative controls were added into relevant wells using jetPRIME® transfection (Polyplus, NY, USA) according to the manufacturer’s instructions. Following transfection for 48–72 h, cells were collected for subsequent experiments.

### Quantitative real time reverse transcription polymerase chain reaction

Total RNA was extracted from different cell lines using Trizol reagent and reverse-transcribed into cDNA with PrimeScript RT Master Mix. The process of qRT-PCR amplification was performed using the Step One Plus Real-Time PCR System (Applied Biosystems, Carlsbad, CA, USA) with FastStart Universal SYBR Green Master. All the experiments mentioned here were performed according to relevant manufacturer’s instructions. The specific primers for human PEG10, E2F-1, and β-ACTIN were designed by Primer Premier 5 and checked by Oligo 6. The formula 2^-ΔΔCt^ (Ct means the cycle threshold) was used to normalize the relative expression of PEG10 mRNA in certain cells.

### Western blotting

Total protein was extracted by using a lysis buffer containing PMSF, protease inhibitors, and phosphatase inhibitors (1 mL lysis buffer with 5 μl 100 mM PMSF, 1 μL protease inhibitors, and 5 μL phosphatase inhibitors). Protein lysates from cells were subjected to 5× SDS-PAGE. Western blot analysis was performed by using standard methods.

### Proliferation assays

#### CCK-8 assay

Cells transfected with siRNA for PEG10 and negative controls were plated in 96-well plates at a density of 2.5 × 10^3^ cells/well. After culturing for 24 h, 100 μL complete medium containing 10 μL CCK-8 reagent was added to each well. Similarly, 100 μL completed medium containing 10 μL CCK-8 reagent was added to respective wells at different time points (24 h, 48 h, 72 h, 96 h, and 120 h). The plates were incubated in dark at 37 °C for 2 h and analyzed at 450 nm absorbance. At least three wells were assessed for each group.

#### Clone formation assay

Different cell lines were seeded in 6-well plates (6 × 10^2^ cells/well). The culture medium was changed every 2 days. The cells were stained with crystal violet after culturing for 10 days. The number of clones was then counted to evaluate cell proliferation.

#### EDU assay

Cells that had undergone different interventions were seeded in 96-well plates at a density of 3 × 10^3^ cells/well. EDU assay was done as per manufacturer’s instructions following 48 h culture. Cell proliferation was assessed by calculating percentage of positive stained cells.

### Apoptosis detection

Cells in each group were harvested after 48 h culture and resuspended in 500 μL Annexin V binding buffer. The cells were stained with 5 μL PE Annexin V and 7-AAD Viability Staining Solution and incubated in dark at room temperature for 15 min before being analyzed by flowcytometer (Gallios, Beckman Coulter, USA).

### Cell cycle detection

Cells transfected with Si-RNA for PEG10 and negative controls were cultured in serum-free medium for 24 h and complete medium for 48 h. Harvested cells were fixed in 70% formaldehyde at 4 °C for 5 h and stained with 500 μL PI/RNase staining buffer. Stained cells were then analyzed by flowcytometer.

### Xenograft tumorigenicity assays

All female BALB/c nude mice aged 5 weeks used in this study were purchased from Model Animal Research Center of Nanjing University. To assess the function of PEG10 in vivo, fresh cancer cells (1 × 10^6^ per mice) were implanted into the subcutaneous tissues which were induced by 1% pentobarbital sodium. The mice were randomly divided into two groups after four weeks of tumor implantation. For each group, intratumoral injection with Si-RNA or negative control was performed once every 48 h. Si-RNA and reagent (in vivo-jetPEI, Polyplus, NY, USA) were separately added in 5% glucose solution, and mixed them together. After a 15 min incubation time at room temperature, the Si-RNA/ in vivo-jetPEI® complexes were injected into animal. After 4 weeks, the mice executed humanely and the tumors were excised and photographed.

### Migration and invasion assays

Cell migration and invasion was assessed using transwell filters purchased from BD Biosciences (Franklin Lakes, NJ). PANC-1 (3 × 10^4^) and CFPAC-1 (3 × 10^4^) cells cultured in serum-free medium for 24 h were seeded into the upper chamber containing an uncoated or Matrigel-coated membrane and 200 μL serum-free medium. Complete medium (500 μL) was added to the lower chamber. Cells that migrated to the lower compartment were stained with crystal violet after 24 h of incubation at 37 °C in a humidified 5% CO2 incubator. Three fields in each well were randomly chosen to count migrated and invaded cells.

### Chromatin immunoprecipitation (ChIP)

ChIP assay was carried out using an EZ-Magna ChIP kit (Millipore, Darmstadt, Germany) according to the manufacturer’s protocol. Briefly, 2 × 10^6^ cells were treated with 1% formaldehyde for 10 min for crosslinking, and then quenched by adding 0.125 M glycine. The cells were scraped with PBS and collected after 5-min centrifugation at 800 × *g* at 4 °C. Then, the cross-linked cells were resuspended in 1% SDS lysis buffer, and the soluble chromatin was sheared into 400-bp fragment DNA using an Ultrasonic Cell Disruptor (Covaris, USA). The fragmented chromatin samples were aliquoted as genomic input DNA or immunoprecipitated with 5 ug E2F-1 antibodies, or rabbit IgG, and incubated at 4 °C with rotation for 16 h. Immunocomplexes collected by magnetic separator were washed and eluted with 1% SDS and 0.1 M NaHCO_3_, and DNA was purified on spin columns. The ChIP products and genomic input DNA were quantitatively analyzed by real-time PCR (E2F-1 primer sequences: forward, 5′- CCTGGAATTATTCTATCTTGCAGAA-3′; reverse, 5′- AATGAATGAAATGCAGCTTTTTAAC-3′). ChIP data are presented as the percentage of input normalized to control purifications.

### Statistical analysis

The paired Student’s *t*-test was applied to compare PEG10 expression in pancreatic cancerous and paired non-cancerous tissues. The association of PEG10 expression with clinicopathologic features was analyzed by the Pearson *χ*2 test. Survival analysis was assessed by Kaplan Meier plots and log-rank tests. Independent prognostic factors were recognized through univariate and multivariate Cox proportional hazard regression models. The comparison between two groups was done by independent Student’s *t*-test. Stata 10.0 software was applied to process survival related analysis, and SPSS 20.0 software were used to perform others. All data were expressed as mean ± SD. Differences were considered statistically significant at *P* < 0.05.

## Results

### The expression and roles of PEG10 in PC

**Fig. 1:**
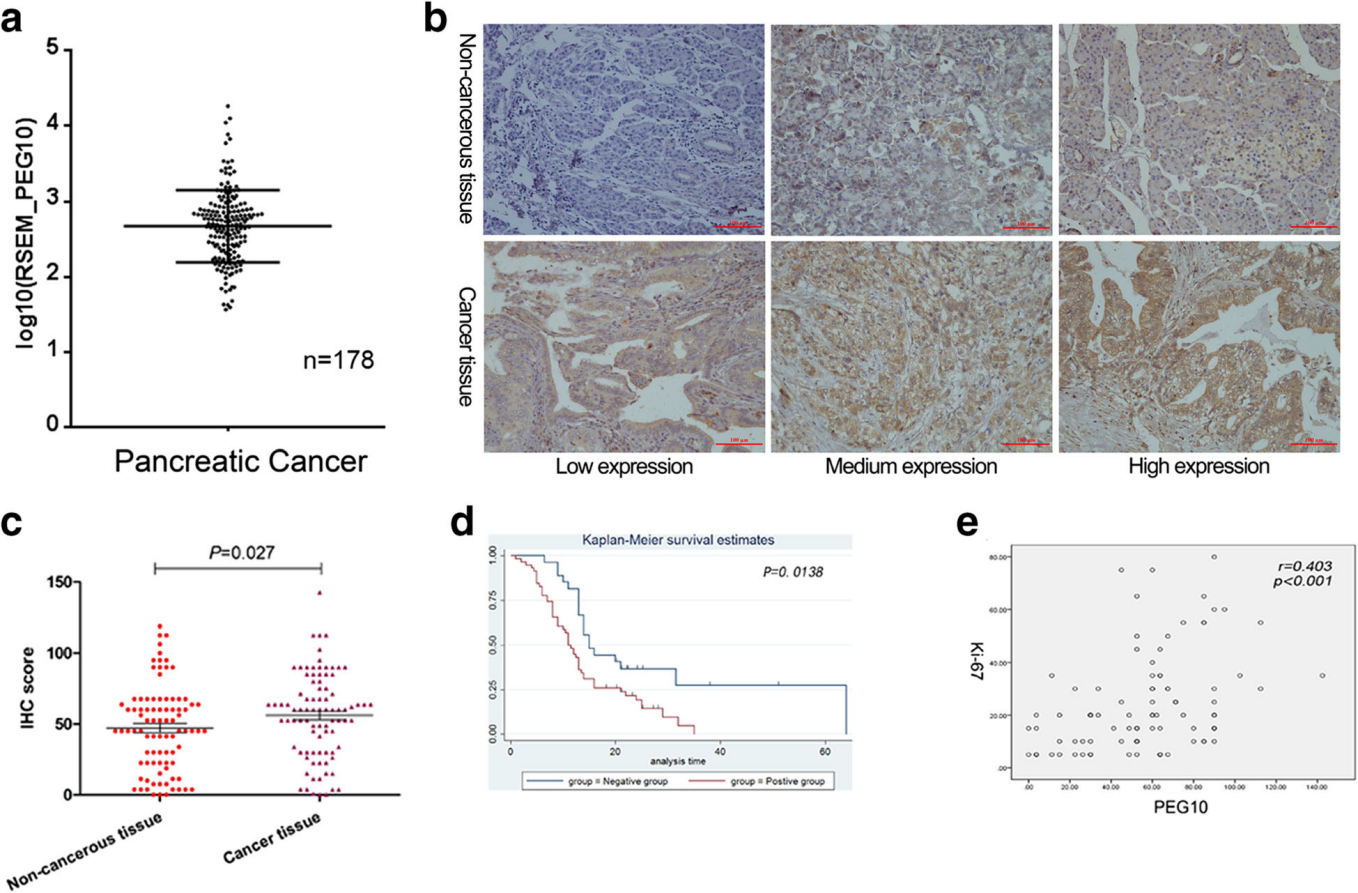
The expression and roles of PEG10 in PC. **a** PEG10 mRNA expression in 178 PC samples obtained from TCGA was shown. **b**, **c** Higher expression of PEG10 was observed in PC tissues than that in non-cancerous tissues by immunohistochemistry. **d** Overexpression of PEG10 was associated with poorer prognosis of PC. **e** The immunohistochemistry scores of Ki-67 were positively correlated with that of PEG10

PEG10 mRNA was generally expressed in PC tissues (*n* = 178) according to the data obtained from TCGA. PEG10 protein detected by IHC was significantly overexpressed in 85 cancerous tissues compared to paired non-cancerous tissues (Fig. 1a-c). Eighteen pairs of tissues were excluded from the analyses because of the absence of target cells in cancerous and/or non-cancerous cases . The association between PEG10 expression and clinicopathological characteristics is depicted in Table 1. High levels of PEG10 in PC were markedly associated with some indicators of PC progression, such as vessel invasion. Further, survival analysis suggested that PC patients with lower expression of PEG10 could have a longer survival time (Fig. 1d). Multivariate analysis suggested that PEG10 was an independent prognostic factor for PC (Table 2). PEG10 expression was positively associated with Ki-67 expression, which is a biomarker of proliferation (Fig. 1e). These data revealed that PEG10 was abnormally upregulated in PC.

**Table 1:** Association of PEG10 expression with the clinicopathological characteristics of PC

Variable	Group	PEG10 expression		*P* value
		High	Low	
CA19-9^a^				*ns*
	≤100	28	17	
	>100	30	10	
Histological grade				*ns*
	I/I-II/II	26	14	
	II-III/III	32	13	
TNM^b^				*ns*
	I-IIA	28	14	
	IIB-IV	30	13	
Lymph node metastasis				*ns*
	Absence	33	16	
	Presence	25	11	
Blood vessel invasion				<0.001*
	Absence	32	22	
	Presence	26	5	
Nerve invasion				*ns*
	Absence	15	11	
	Presence	43	16	

*CA19-9* ^a^ carbohydrate antigen 19–9, *TNM* ^b^ tumor-node-metastasis

*P < 0.05

**Table 2:** Univariate and multivariate analysis of the association of prognosis with clinicopathologic parameters and PEG10 expression in PC

Variable	Univariable analysis		Multivariable analysis	
	HR^a^ (95% CI^b^)	*P*	HR (95% CI)	*P*
CA19-9 (≤100 *vs.* > 100)	1.49 (0.92–2.42)	0.107	1.15 (0.70–1.90)	0.587
Histological grade (I/I–II/II *vs.* II–III/III)	1.90 (1.16–3.11)	0.011*	1.96 (1.17–3.30)	0.011*
TNM (I–IIA *vs.* IIB–IV)	1.28 (0.81–2.03)	0.298		
Lymph node metastasis (Absence *vs.* Presence)	1.17 (0.72–1.91)	0.515		
Blood vessel invasion (Absence *vs.* Presence)	1.96 (1.20–3.19)	0.007*	1.90 (1.14–3.15)	0.014*
Nerve invasion (Absence *vs.* Presence)	1.47 (0.86–2.54)	0.162	1.25 (0.71–2.21)	0.442
PEG10 expression (Low *vs.* High)	1.93 (1.12–3.34)	0.018*	1.85 (1.06–3.21)	0.030*

^a^ *HR* hazard ratio, ^b^ *CI* confidence interval

*p < 0.05

### Inhibition of PEG10 following Si-RNA transfection in CFPAC-1 and PANC-1 cells

**Fig. 2:**
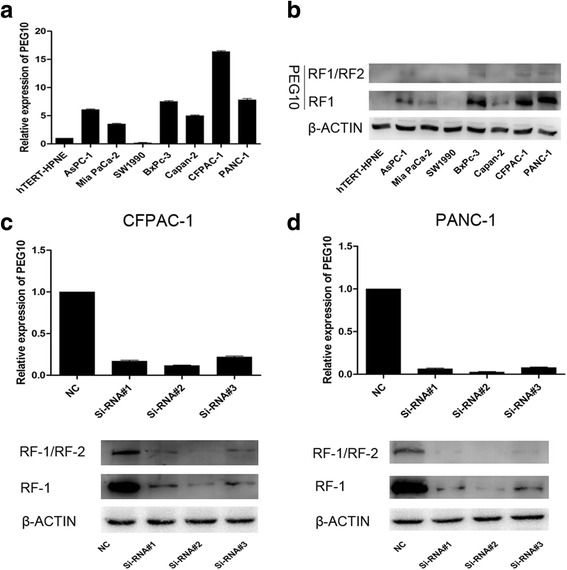
The interference efficiency of three Si-RNAs for PEG10. **a**, **b** The mRNA and protein expression of PEG10 in different PC cell lines were shown. **c**, **d** The interference efficiency of three Si-RNAs for PEG10 was confirmed through both RT-PCR and western blotting

The expression of PEG10 was detected in AsPC-1, Mia PaCa-2, SW1990, BxPc-3, Capan-2, CFPAC-1, PANC-1, and hTERT-HPNE cells by qRT-PCR and western blotting. Compared to hTERT-HPNE cells, higher expression of PEG10 mRNA and protein were observed in PC cells, especially CFPAC-1 and PANC-1 (Fig. 2a and b). Therefore, CFPAC-1 and PANC-1 cells were selected to conduct Si-RNA related assays.

The interference efficiency of three Si-RNAs for PEG10 was confirmed through comparison with negative controls at both mRNA and protein levels (Fig. 2c and d). Si-RNA#2 could significantly decrease the production of PEG10 and was chosen for further functional and mechanistic analyzes.

### Si-RNA induced PEG10 downregulation suppresses PC cell proliferation by arresting cell cycle in G0/G1 phase

Since PEG10 expression was positively associated with Ki-67 expression, we further investigated whether PEG10 could affect PC cell proliferation.

**Fig. 3:**
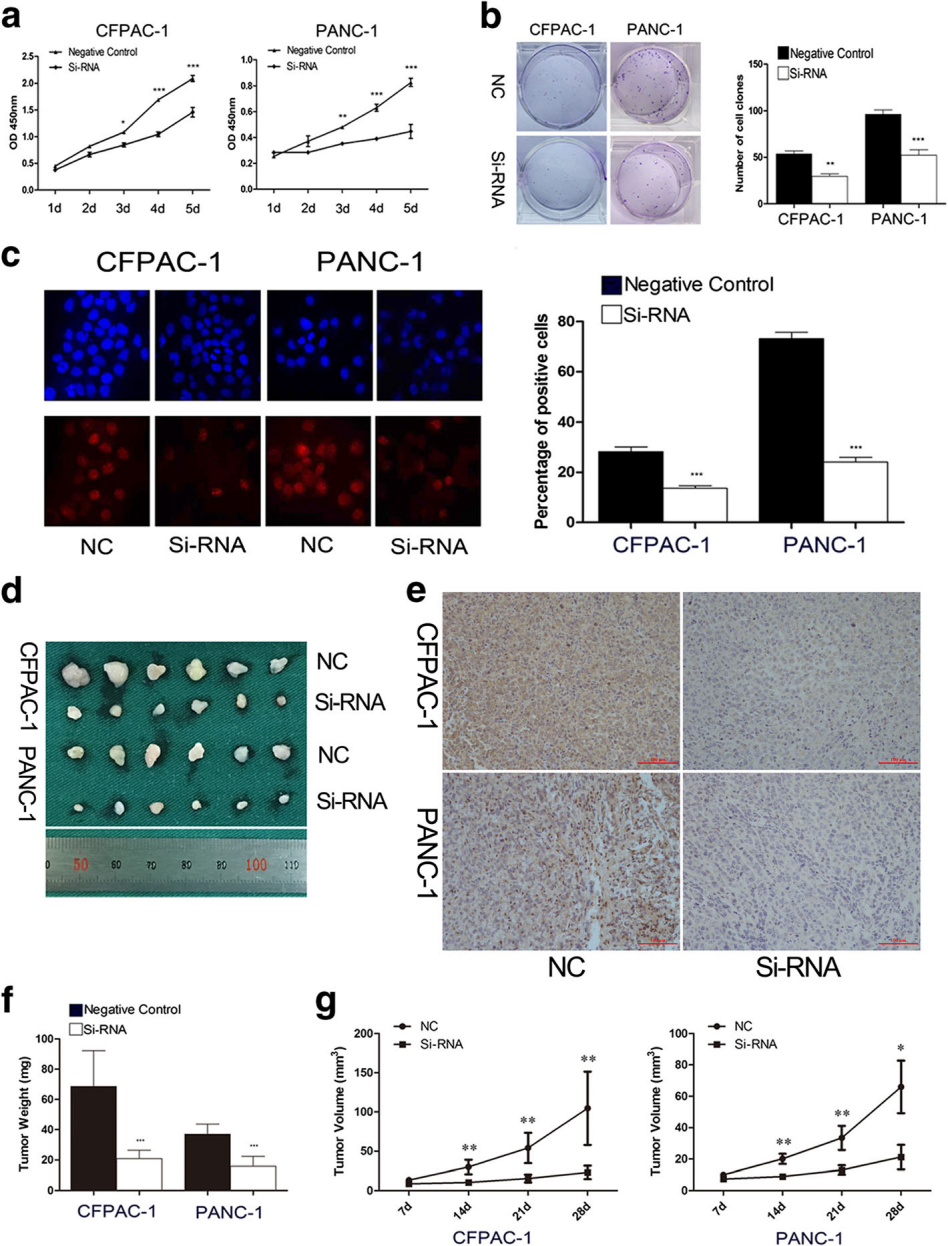
The negative effects of PEG10 knockdown on PC cell proliferation. **a**-**c** The proliferation of PC cells was decreased following PEG10 downregulation by Si-RNA in vitro. **d**, **e** The expression of PEG10 was decreased after PEG10 knockdown in vivo. **f**, **g** The weight and volume of tumors obtained from animal models were decreased following PEG10 downregulation by Si-RNA in vivo. ^*^ represents *P* < 0.05,^**^ represents *P* < 0.01, and ^***^ represents *P* < 0.001

Simultaneously, CCK-8, clone formation, and EDU assays were used to investigate the influence of PEG10 on PC cell proliferation. The results of CCK-8 assay demonstrated that the proliferation of Si-RNA transfected cancer cells was markedly reduced compared to negative control transfected cells (Fig. 3a). The number of cell clones in PEG10 downregulated cells was more than that in control cells (Fig. 3b). EDU results also show similar result with the percentage of positive stained cells being significantly decreased in interference groups (Fig. 3c). The interference efficiency of Si-RNA for PEG10 in vivo was further confirmed by using IHC (Fig. 3d and e). Furthermore, the volume and weight of the tumors obtained from animal models injected with Si-RNA were both lower than that treated with negative controls (Fig. 3f and g). This data suggests that PEG10 could promote PC cell proliferation in vitro and vivo.

Although we found that PEG10 could promote PC cell proliferation, the mechanisms involved in this phenomenon were still unclear. It is generally considered that cell apoptosis enhancement and/or cell cycle arrest may inhibit the proliferation [16, 17]; however, whether these processes contributed to mediation of PC cell proliferation by lowering the expression of PEG10 is not known.

**Fig. 4:**
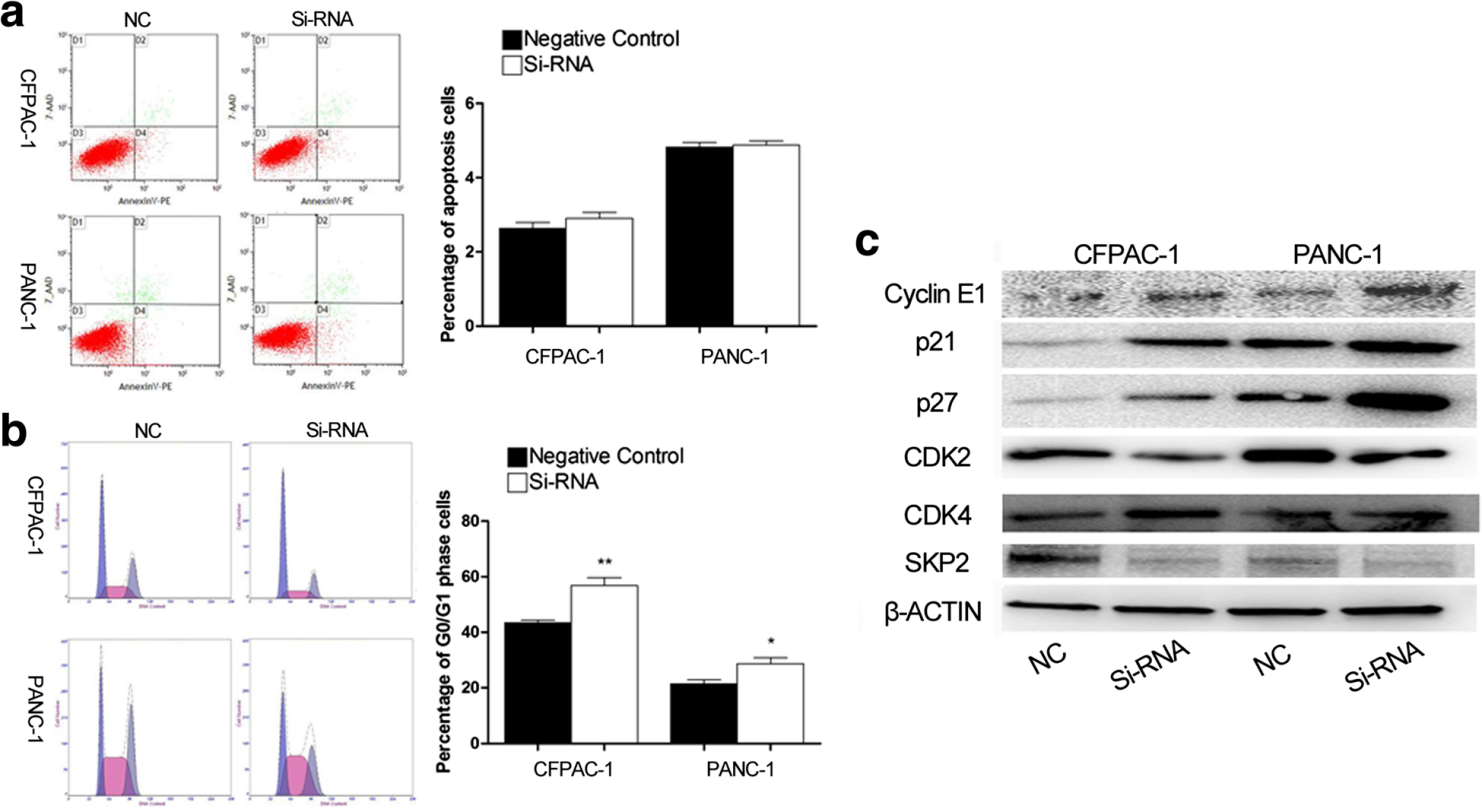
The influence of PEG10 knockdown in PC cells on G0/G1 arrest. **a** The percentage of PC cell apoptosis was similar in each group. **b**, **c** Downregulation of PEG10 in PC cells induced G0/G1 arrest through increasing the production of p21 and p27. ^*^ represents *P* < 0.05,^**^ represents *P* < 0.01, and ^***^ represents *P* < 0.001

As shown in Fig. 4a, the percentage of apoptotic cells had no significant statistical differences between groups showing PEG10 downregulation and negative control groups. However, the percentage of G0/G1 phase cells in PEG10 interfered cells was significantly higher than that in control cells (Fig. 4b). This result indicated that downregulation of PEG10 could induce G0/G1 phase arrest to further suppress cell proliferation in PC.

To investigate how downregulation of PEG10 induces G0/G1 phase arrest, the levels of several proteins which play crucial roles in G0/G1 phase were detected by western blotting. Cyclin E1 is reported to accumulate from G0/G1 to S phase and reduce steadily from S to G2/M phase. The abundant accumulation of this molecule in G1/G0 generally indicates G0/G1 arrest. Our results suggested that the levels of Cyclin E1 were significantly higher in PEG10 knockdown PC cells than that in control cells (Fig. 4c). Furthermore, two regulators of cell cycle progression at G1 phase, p21 and p27, were markedly upregulated following PEG10 knockdown. The production of CDK2 which is negatively regulated by p21 and p27 was significantly downregulated after PEG10 silencing (Fig. 4c). Moreover, the expression of SKP2 was downregulated following PEG10 silence (Fig. 4c). These data suggested that interference of PEG10 in PC cells could induce G0/G1 arrest by upregulation of p21 and p27.

### PEG10 promotes the migration and invasion of PC cells through ERK/MMP7 pathway

Since higher PEG10 expression was significantly associated with vessel invasion in PC samples, we speculated that PEG10 may promote the migration and invasion of PC cells.

**Fig. 5:**
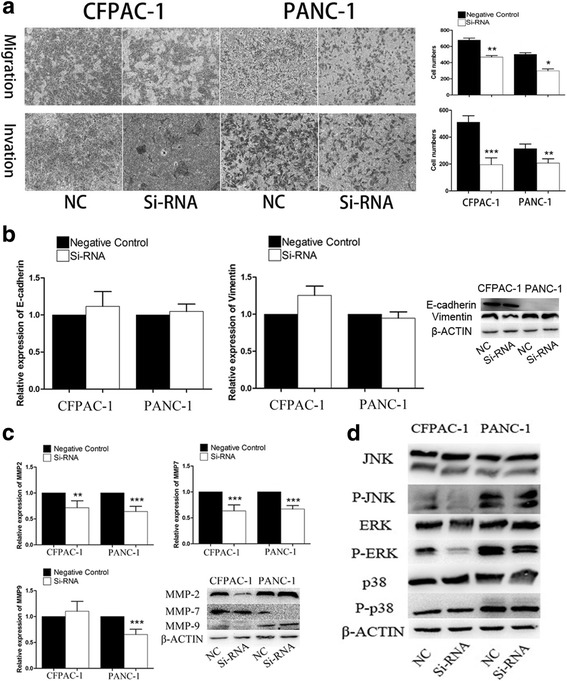
The negative effects of PEG10 knockdown on migration and invasion of PC cells. **a** The migration and invasion of PC cells was decreased in PEG10 downregulated groups. **b** The expression of EMT markers remained unchanged between two groups. **c**, **d** PEG10 triggered the migration and invasion of PC cells through ERK/MMP7 pathway. ^*^ represents *P* < 0.05,^**^ represents *P* < 0.01, and ^***^ represents *P* < 0.001

As shown in Fig. 5a, the migration and invasion of PC cells was significantly decreased following the downregulation of PEG10. We further investigated the expression of epithelial-mesenchymal transition (EMT) markers and matrix metalloproteinases (MMPs) that are widely reported to mediate the migration and invasion of cancer cells. Our results demonstrated that expression of EMT markers was similar in different groups, but MMP7 expression was markedly reduced in PEG10 knockdown cells (Fig. 5b and c). Although the mRNA of MMP2 was both decreased in two pancreatic cancer cell lines after the silence of PEG10. However, the protein of MMP2 was only decreased in CFPAC-1. Further, we observed that reduction in phosphorylation of ERK may be responsible for the downregulation of MMP7 (Fig. 5d). These findings suggest that ERK/MMP7 pathway may mediate the PEG10 induced migration and invasion of PC cells.

### E2F-1 directly regulates PEG10 expression

**Fig. 6:**
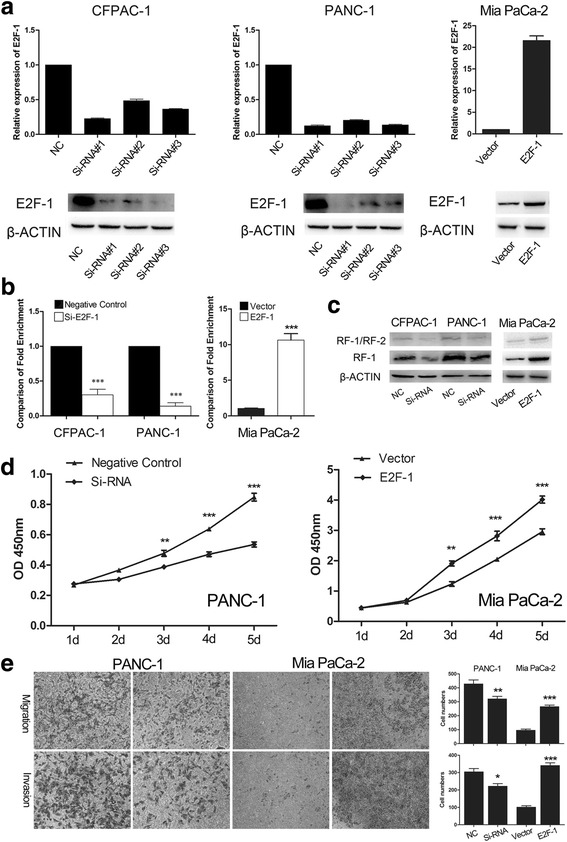
Direct regulation of transcription factor E2F-1 on PEG10 expression. **a** The interference efficiency of three Si-RNAs and plasmid vector for E2F-1 was confirmed through both RT-PCR and western blotting. **b**, **c** E2F-1 could bind to the promoter of PEG10, and the binding efficiency and PEG10 protein expression was decreased or increased in E2F-1 knockdown or overexpression groups. **d**, **e** The proliferation, migration, and invasion of PC cells was affected after E2F-1 knockdown or overexpression. ^*^ represents *P* < 0.05,^**^ represents *P* < 0.01, and ^***^ represents *P* < 0.001

PEG10 is reported to be directly regulated by E2F-1 in hepatocellular carcinoma and neuroendocrine prostate cancer [18, 19]. However, whether E2F-1 regulates the expression of PEG10 in PC cells was not known. To evaluate we conducted ChIP assay and the results demonstrate that E2F-1 could bind to the promoter of PEG10 and the binding efficiency was respectively decreased or increased in E2F-1 knockdown or overexpression (Fig. 6a and b). The expression of PEG10 was also decreased or increased in E2F-1 knockdown or overexpression cells at protein levels (Fig. 6c). Furthermore, the proliferation, migration, and invasion of PC cells were respectively inhibited or promoted after E2F-1 knockdown or overexpression (Fig. 6d and e). Data here suggested that E2F-1 could directly regulate PEG10 expression to further affect the cell proliferation, migration, and invasion in PC cells.

## Discussion

In this study, we show that PEG10 was abnormally overexpressed in PC and was significantly associated with PC progression and prognosis. Furthermore, E2F-1 mediated PEG10 upregulation could promote PC cell proliferation, migration, and invasion. Thus, we identified PEG10 as a potential prognostic and therapeutic target for PC.

PEG10 is a paternally expressed imprinted gene, which was reported to have been derived from the Ty3/Gypsy family of retrotransposons [20]. Similar to many other imprinted genes, the function of PEG10 is decided by its expression levels or methylation level [21–23]. In our study, we focused on the altered expression of PEG10 in PC. During physiological conditions, adequate PEG10 expression is necessary for placental development and it has been shown that PEG10 is downregulated in case of preeclampsia [24–26]. PEG10 expression was later shown to be abnormally reactivated in some malignancies (such as liver cancer, lung cancer, and gallbladder cancer) and that it could be a biomarker for progression and prognosis of certain cancers [13, 27, 28]. The findings of our study show for the first time that PEG10 is highly expressed in PC and is associated with tumor size, vessel invasion, and shorter overall survival time. Cox proportional hazard regression analysis further illustrates that PEG10 is an independent factor for poor prognosis of PC.

Furthermore, we also propose the role of PEG10 in PC cell proliferation and the underlying mechanism. PEG10 has been reported to promote cancer cell proliferation through mechanisms described as follows. PEG10 overexpression was reported to be associated with a mediator of apoptosis (SIAH1) resulting in decrease in cell death as in hepatocellular carcinomas [12]. In neuroendocrine prostate cancer, PEG10 knockdown was shown to suppress cell cycle progression through upregulation of p21 and p27 and downregulation of CDK2 [19]. In the present study, we also investigated the proliferation promoting effect of PEG10 considering these two mechanisms; and the results were similar to the previously published studies. Cell apoptosis rate in PEG10 knockdown PC cells was similar to that in control cells. This phenomenon may due to the inefficiency of PEG10 mediated anti-apoptosis effect or the presence of other apoptosis regulatory mechanisms. However, the G0/G1 phase arrest in PEG10 knockdown PC cells was obviously observed. Further molecular mechanism analysis suggested that expression of p21 and p27, which are known cyclin-dependent kinase inhibitors, were significantly increased in cells with PEG10 downregulation. These molecules could inhibit the activation of CDK2 and CDK4 through binding to them, as reported previously [29, 30]. However, the expression of CDK4 was not significantly affected in cells with PEG10 downregulation and control cells. Furthermore, the expression of SKP2 was negatively associated with p27 which was similar to the results of previous studies in pancreatic cancer [31, 32]. Data related to cell cycle were consistent with the studies described above.

Though the roles of PEG10 in PC cell proliferation, apoptosis, and cell cycle have been investigated in the present study, the roles of this protein in other aspects are relatively unknown. In previous studies, it has been reported that PEG10 could induce migration of Burkitt’s lymphoma cells via upregulation of MMP-2 and MMP-9 [33]. PEG10 has been reported to promote lung cancer cell migration and invasion by upregulating the expression of β-catenin, MMP-2 and MMP-9, and decreasing the expression of E-cadherin [13]. Further, PEG10 was shown to trigger prostate cancer cell invasion by enhancing Snail expression via TGF-β signaling [19]. Our findings show that PEG10 expression is positively associated with vessel invasion in PC, and PEG10 promotes PC cells migration and invasion through ERK/MMP7 pathway. The MMP2 mRNA was both decreased in two pancreatic cancer cell lines after PEG10 knockdown. However, the protein of MMP2 was only decreased in one of the cell lines (CFPAC-1). Therefore, these data was not sufficient to suggest that MMP2 was regulated by PEG10.

Although downstream events of PEG10 in PC have been discussed preliminarily, the upstream events of this protein are still unclear. E2F-1 is a member of the E2F family of transcription factors, which plays a crucial role in the control of cell cycle. E2F-1 has been reported to regulate PEG10 expression via directly binding to the promoter of PEG10 in liver and prostate cancer [18, 19]. Similar to these studies, our ChIP and western blotting assay results also demonstrated that E2F-1 could interact with PEG10 promoter to further upregulate PEG10 expression. Furthermore, PEG10 could also be regulated by some other factors, such as c-MYC, CXCL13, CCL19, and androgen receptor. Li et al. revealed that c-MYC, which serves as a classic proto-oncogene, could act directly upstream of proliferation-positive gene PEG10 [10]. Levels of PEG10 expression in freshly isolated B-ALL and B-CLL CD19^+^CD34^+^ B cells has been shown to be significantly increased after stimulation with CXCL13 and CCL19 together [34]. The upregulation of PEG10 in this pattern may be involved in the resistance to TNF-α-mediated apoptosis of B cells. Moreover, the interaction between androgen and androgen receptor was firstly described as a facilitating factor for PEG10 expression in hepatic cancer [35], and then confirmed in gastric cancer [36]. Whether the factors discussed above participate in the activation of PEG10 in PC needs to be further investigated.

## Conclusions

We have demonstrated that an increased expression of PEG10 is associated vessel invasion, and the overall survival time of PC patients. E2F-1 mediated PEG10 overexpression promotes PC cell proliferation via accelerating G0/G1 progression and increase migration and invasion through ERK/MMP7 pathway. These results suggest that PEG10 may serve as an oncogene in PC pathogenesis and is a potential prognostic and therapeutic target for PC.
